# Yield of the Four-Carbon Stabilized Criegee Intermediates from Isoprene Ozonolysis

**DOI:** 10.1021/acsearthspacechem.5c00226

**Published:** 2026-01-08

**Authors:** Rabi Chhantyal-Pun, Pengcheng Wang, Shefali Baweja, Joseph Bainbridge, Chenyang Xue, Véronique Daële, Abdelwahid Mellouki, Max R. McGillen

**Affiliations:** † School of Chemistry, 6123 University of Nottingham, Nottingham, NG7 2RD, U.K.; ‡ CNRS, 57495 Institut de Combustion Aérothermique Réactivité et Environnement (ICARE), Orléans, 45071, France; § 479571 Mohammed VI Polytechnic University, Ben Guerir 43150, Morocco

**Keywords:** Isoprene, Ozonolysis, Criegee Intermediates, methyl vinyl ketone oxide, methacrolein oxide

## Abstract

Isoprene is the single most abundant nonmethane hydrocarbon emitted into the atmosphere. Despite this, uncertainties in the oxidation chemistry remain. Here, we investigate the yields of Criegee intermediates that are produced from the ozonolysis reaction, where we conduct a series of atmospheric simulation chamber experiments in which the transient stabilized Criegee intermediates (sCIs) are titrated in the gas phase using either biacetyl or acetylpropionyl. This reaction yields a stable ketone-substituted secondary ozonide (SOZ), which was observed directly in the gas phase using a proton-transfer-reaction time-of-flight mass spectrometer operated in NH_4_^+^ mode. Both C_1_ and C_4_ sCIs were observed in this way, with the mass of the NH_4_^+^ adduct shifting according to the mass of the sCI and its diketone titrant. The relative abundance of the C_4_ sCI was constrained against C_1_ assuming a similar sensitivity for the two SOZ derivatives. This was supported by quantum chemical calculations that demonstrated very similar binding energies between NH_4_^+^ and the C_1_ and C_4_ SOZ adducts. Our results demonstrate an overall yield of ∼11% for the long-lived C_4_ sCIs, which may survive long enough to participate in various bimolecular reactions in the atmosphere.

## Graphical abstract

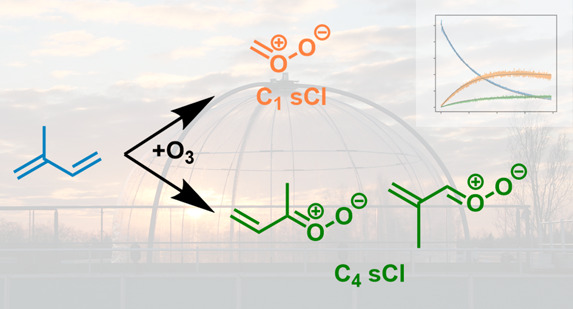

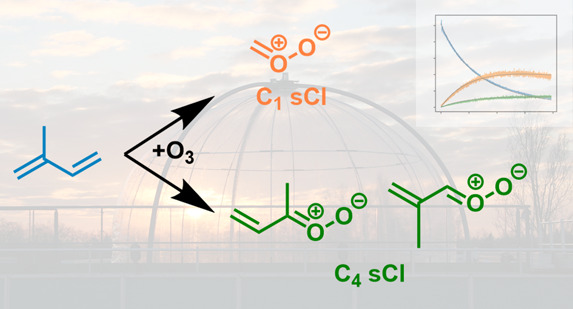

## 1 Introduction

Total emissions of isoprene amount to between 378 and 496 Tg C a^–1^.^1^ This large mass flux paired with its high reactivity leads to potent effects on oxidizing capacity, secondary organic aerosol (SOA) formation, particle nucleation, and NO_*x*_ partitioning. Accurate degradation mechanisms are therefore of major importance to global chemical modeling.^2^ Isoprene is reactive toward hydroxyl (OH), ozone (O_3_), and nitrate (NO_3_),^3^ and under clear sky conditions, O_3_ accounts for ∼ 10% of the total oxidation budget.^2^ However, isoprene is short-lived, and much of it will be consumed within the canopy, where shading suppresses OH production.^4,5^ Consequently, alkene degradation by O_3_ can become competitive with OH in forested environments.^6^ Here, we focus on the reactions between isoprene and ozone.

Isoprene–ozone reactions have been studied previously, and stable end-product yields are now well-established.^7,8^ The fate of the stabilized Criegee intermediates, sCIs, produced in these reactions has been studied most notably by Nguyen et al.^9^ which constituted a large, interdisciplinary effort to resolve the fates of these elusive species, concluding that the dominant fate of the one-carbon sCI would be reaction with water vapor, whereas the four-carbon sCIs would be lost through unimolecular decomposition. Nevertheless, significant progress has been made in both the study of sCIs and analytical chemistry in recent years. First, the emergence of clean and convenient sCI syntheses employing *gem*-diiodoalkyl precursors has led to the first direct detection of an sCI using photoionization,^10^ followed by subsequent measurements in the UV and IR.^11,12^ This prompted a large number of kinetic measurements, which has greatly expanded our knowledge of the unimolecular and bimolecular reactions affecting sCIs.^13,14^ Second, the increased use of soft-ionization techniques paired with high-resolution mass spectrometry has greatly expanded the selection of compounds that can be detected with high sensitivity and mass speciation.^15^ Here, we utilize these recent developments in kinetics and analytical chemistry to conduct experiments in a state-of-the-art chamber facility,^16^ where we will demonstrate the unambiguous detection of derivatized four-carbon stabilized Criegee intermediates from isoprene ozonolysis for the first time, suggesting that previous studies underestimate the potential of larger sCIs to participate in bimolecular processes in the atmosphere.

Previous chamber and flow tube studies have used SO_2_ or ketones such as hexafluoroacetone to scavenge Criegee intermediates and find the yield of stabilization.^17–20^ These studies were able to measure the total yield of the stabilized Criegee intermediates. Some studies have used H_2_O, specifically (H_2_O)_*n*_ where *n* ≥ 2, to selectively scavenge the one-carbon stabilized Criegee intermediate, C_1_ sCI, by detecting the hydroxymethyl hydroperoxide product and suggest a high yield of ∼ 0.6.^9,21^ More recent work by Nguyen et al. suggested that the yield of the four-carbon stabilized Criegee intermediate, C_4_ sCIs, is insignificant from their chamber study using SO_2_ as a scavenger.^22^ However, studies by Sipila et al., Newland et al. and Rickard et al. all observed indirect evidence for at least two different types of sCIs based on reactivity with H_2_O, SO_2_, and DMS suggesting significant yield of the C_4_ sCIs.^17,18,23^ C_4_ sCIs are estimated to be the dominant Criegee intermediates in the atmosphere by various modeling studies based on unimolecular and bimolecular reactivity studies.^24–26^ Therefore, a central aim of this study is to resolve this apparent discrepancy between Nguyen et al. and the indirect studies, such that chemical modeling of forested regions can be improved.

Spectroscopic measurements and computational studies have shown that there are four main isomers of C_4_ sCI *syn-*methyl vinyl ketone, *syn-*MVKOO, *anti-* methyl vinyl ketone, anti-MVKOO, *syn-*methacrolein oxide, *syn-*MACROO, and *anti-*methacrolein oxide, *anti-*MACROO as shown in Figure 1.^25,27,28^ All of these isomers also have E and Z isomers based on the orientation of the vinyl group, but those have fast interconversion rates. The *anti-*MVKOO and *syn-*MACROO isomers are predicted to undergo fast unimolecular reactions to produce dioxoles.^25,29,30^ The *syn-*MVKOO and *anti-*MACROO isomers have been shown to undergo slow unimolecular reactions and react slowly with H_2_O.^28,31,32^ Based on these observations and predictions, atmospheric modeling studies predict *syn-*MVKOO and *anti-*MACROO species to be the dominant sCIs in the troposphere.^26^

**Figure 1:**
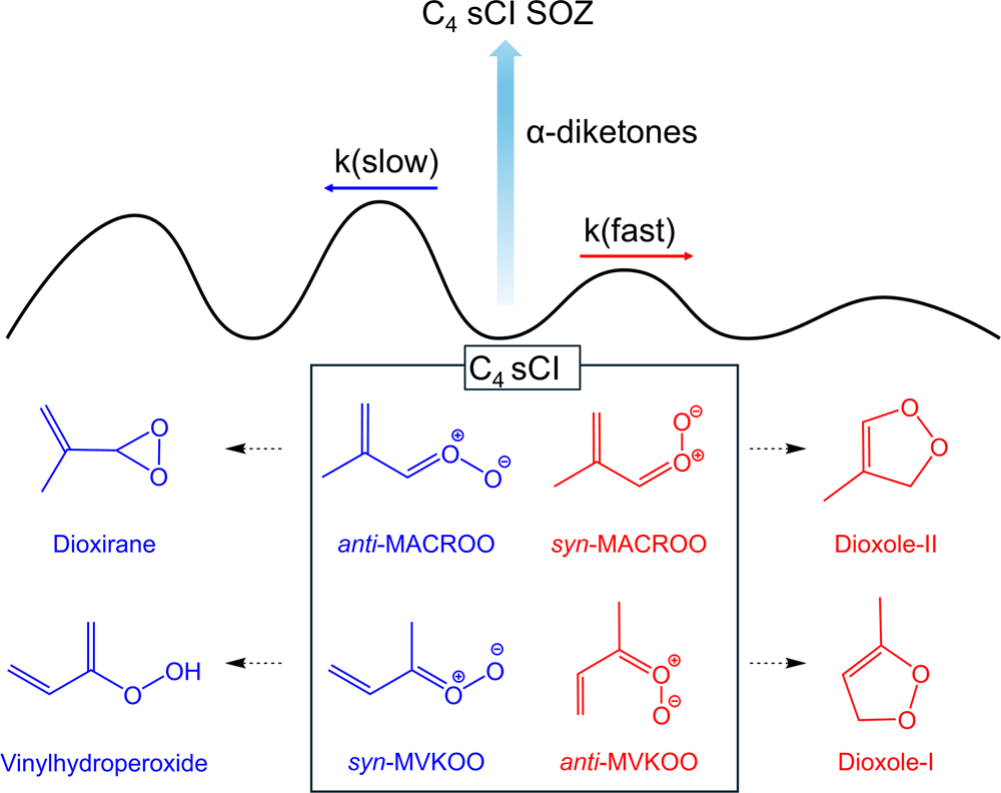
Various isomers of the four-carbon stabilized Criegee intermediates, C_4_ sCI, produced during isoprene ozonolysis. Scavenging reactions of α-diketones used in this study compete with unimolecular isomerization reactions.

Recently, laser flash photolysis measurements by Cornwell and co-workers have shown that C_1_ sCI reacts rapidly with α-diketones such as biacetyl and acetylpropionyl, ∼ 10^–11^ cm^3^ s^–1^, and predict the formation of secondary ozonide adducts.^33^ The combination of large rate coefficients with Criegee intermediates and production of a stable secondary ozonide (SOZ) adduct, as shown in Scheme 1, indicates that α-diketones are highly effective scavengers for quantitative derivatization of Criegee intermediates. SOZs from reactions of sCIs and simple ketones have been observed previously using FTIR in chamber and ambient pressure flow tube studies.^19,34,35^ In this study, the Criegee intermediates generated during ozonolysis of isoprene under ambient conditions were scavenged using biacetyl or acetylpropionyl, and the derivatized SOZ adducts were detected quantitatively using NH_4_^+^ chemical ionization mass spectrometry to estimate the mass speciated stabilization yields of the sCIs.

**Scheme 1:**
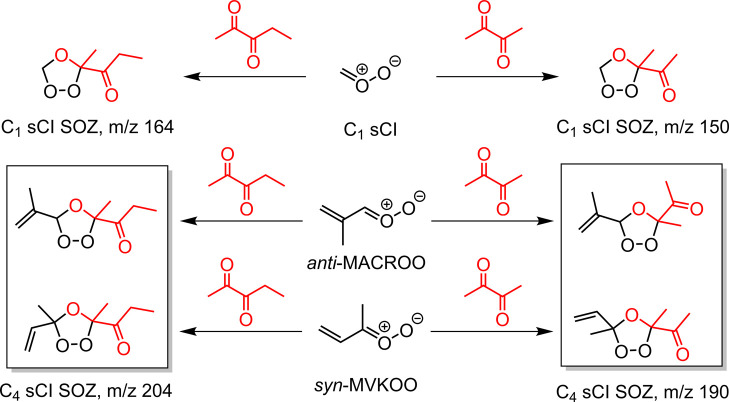
Scavenging Reactions of Biacetyl and Acetylpropionyl with the One- and Four-Carbon Stabilized Criegee Intermediates, C_1_ sCI, and C_4_ sCI (*syn*-MVKOO and *anti*-MACROO), to Form Respective Derivatized Secondary Ozonide, SOZ Adducts^a^

a Also shown are the m/z values for the cluster of the SOZ adducts with an NH_4_^+^ ion.

## 2 Methods

### 2.1 Atmospheric Simulation Chamber

Experiments were conducted in the HELIOS chamber, which is described in detail in a previous publication.^16^ In brief, HELIOS is a 90 m^3^ hemispheric atmospheric simulation chamber constructed of 125 μm thick FEP Teflon foil. The chamber contents are maintained at a slight overpressure using a small flow of zero air (50 SLM) in order to prevent the introduction of contaminants into the atmosphere. Approximately 70 ppb of SF_6_ is added to the chamber at the beginning of each experiment, which is continuously monitored using a multipass (path length = 303 m) FTIR spectrometer (Vertex 70, Bruker) in order to assess the dilution of the chamber composition caused by this zero-air flow. Reactive mixtures are stirred continuously using two fans positioned at the chamber base, ensuring that chamber contents become homogeneous within a ∼ 2 min time frame, with overall experimental durations of several hours. Ozone is introduced to the chamber using a high-concentration ozone generator (BMT 803 BT) and is analyzed using an ozone monitor (49i, Thermo Fisher Scientific). Organic molecules (e.g., isoprene, dicarbonyls, SOZ molecules) were monitored continuously using a PTR-ToF-MS (PTR-8000, IONICON) that was operated in NH_4_^+^ mode,^36^ providing good sensitivity to oxygenated molecules, while limiting ion fragmentation through low drift-tube voltages (220 V, E/N = 46.3 Td). The mass spectra were calibrated based on the *m*/*z* signals at 29.998 (NO^+^), 69.0704 (protonated isoprene), and 83.0861 (protonated cyclohexene). The OH radical chemistry was suppressed using high concentrations (30.6 ppm) of cyclohexane. Because of the potentially lossy nature of peroxidic species to surfaces (especially the catalytic losses presented by metallic surfaces), chamber contents were sampled through a purpose-built high-throughput (∼15 SLM) sampling manifold in which all wetted surfaces were either of Pyrex or PFA Teflon, as described previously.^37^

### 2.2 Quantum Chemistry Calculations

The potential energy surface of SOZs derived from C_1_ sCI and C_4_ sCIs (*syn*-MVKOO and *anti*-MACROO) with biacetyl, as well as their complexes with NH_4_^+^, were investigated using the GFN-xTB method within the CREST (Conformer-Rotamer Ensemble Sampling Tool) framework.^38^ This approach employs a semiempirical tight-binding method coupled to metadynamics for efficient conformational sampling. The resulting structures for the C_1_ sCI and C_4_ sCI derived SOZs and the complexes of these SOZs with NH_4_^+^ ions were further optimized at the B3LYP-D3BJ/6–311++G­(d,p) level of theory using Gaussian16.^39^ Harmonic frequency calculations were also performed for the optimized structures to confirm true minima and to provide zero-point-energy-corrected binding energies.

## 3 Results

### 3.1 Derivatization of Stabilized Criegee Intermediates

Isoprene ozonolysis experiments were performed in the HELIOS chamber in the presence of α-diketones, biacetyl or acetylpropionyl or methanol and cyclohexane (an OH scavenger).^37^ Reactions were initiated by the injection of isoprene after all the other chemical and physical conditions had stabilized in the chamber, typically 1–2 h duration. Table 1 summarizes the experiments performed for this study.

**Table 1:** Summary of Experiments Performed during This Study^a^

**Exp**	**Scavenger**	**[Scavenger]**	**[Isoprene]**	**[Ozone]**	**[Cyclohexane]**
I	Methanol	1.6 × 10^14^	2.6 × 10^13^	5.8 × 10^13^	7.4 × 10^14^
II	Acetylpropionyl	1.0 × 10^14^	2.6 × 10^13^	6.4 × 10^13^	7.4 × 10^14^
III	Biacetyl	8.3 × 10^13^	2.6 × 10^13^	6.3 × 10^13^	7.4 × 10^14^
IV	Biacetyl	4.1 × 10^13^	2.6 × 10^13^	5.8 × 10^13^	7.4 × 10^14^

a All initial concentrations are provided in units of molecules cm^–3^. The chamber pressure and temperature were ∼ 740 mbar and ∼ 293 K for all the experiments performed during this study.

Figure 2a-d shows the mass spectra (10s integration) obtained 1000 s after the initiation of the ozonolysis experiments using methanol, acetylpropionyl, and biacetyl scavengers. Signal intensities were scaled with respect to the isoprene concentration used in the various experiments. This enabled direct comparison of any changes in mass signals between the four experiments. Using methanol as a scavenger did not result in distinct adduct signals for C_1_ sCI and C_4_ sCI, likely a consequence of the much lower reactivity of methanol, resulting in insufficient scavenging rates to compete with unimolecular loss processes. The *m*/*z* signals at 150, 190 and 164, 204 are enhanced significantly in the α-diketone scavenging experiments. These masses are consistent with the C_1_ sCI and C_4_ sCI SOZ adducts with the α-diketones as shown in Scheme 1 complexed with the NH_4_^+^ ion reagent. No significant signals were observed at the protonated SOZ masses. A small interfering signal around *m*/*z* 150 was observed in experiments using either the two α-diketones or methanol scavengers, as shown in Figure 2. Thus, this background signal likely arises from the ozonolysis experiment rather than the scavenger chemistry and was subtracted before performing kinetic analysis for the biacetyl experiments as discussed in the next section. The background signal at the other masses was relatively small, and no background subtraction was performed. All SOZ signals showed steady decreases in intensity with increasing drift tube electric field strength, ranging from 30 to 105 Td, indicating complexation with NH_4_^+^ as shown in Figures S1 and S2 in the Supporting Information. The signals at *m*/*z* 190 and 204 decreased when O_3_ concentration was increased after the end of the kinetic experiments as shown in Figures S3 and S4, indicating the presence of an olefinic bond. At the same time, the signals at *m*/*z* 150 and 164 increased likely because of the increase in C_1_ sCI production from ozonolysis of products such as methyl vinyl ketone and methacrolein. These observations confirm the assignment of signals at *m*/*z* 150 and 164 to C_1_ sCI SOZ and signals at *m*/*z* 190 and 204 to C_4_ sCI SOZ.

**Figure 2:**
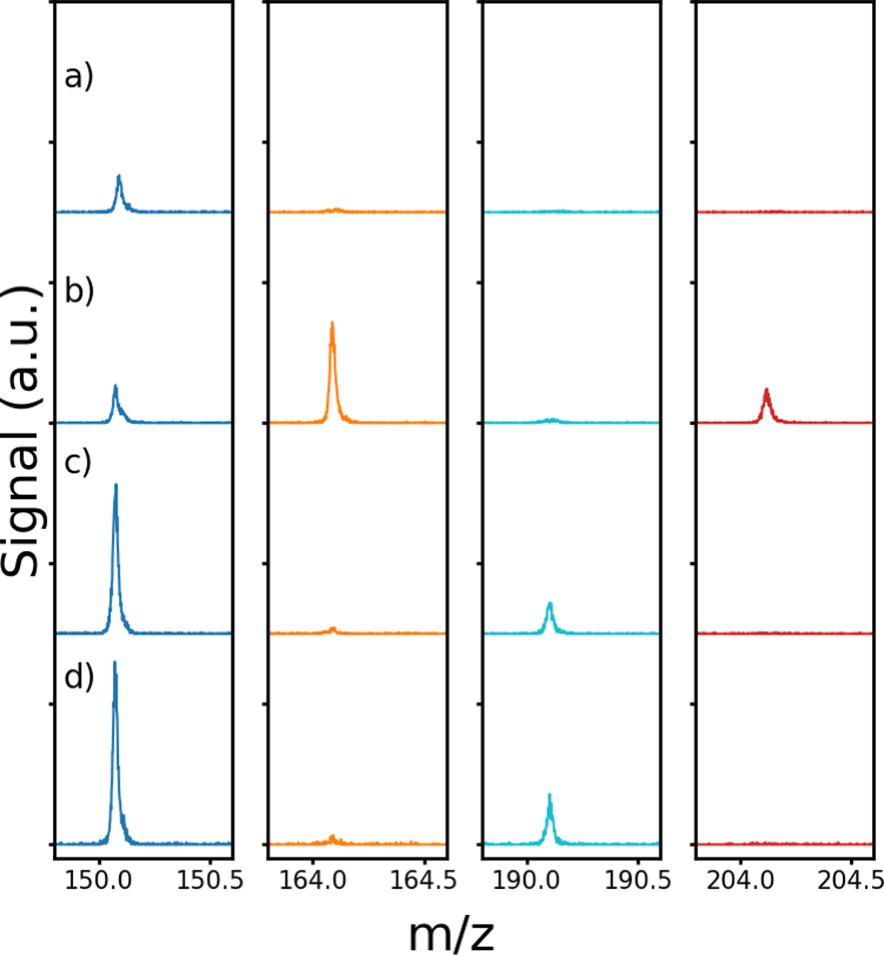
Mass spectra from isoprene ozonolysis in the presence of a) methanol (Exp I), b) acetylpropionyl (Exp II), c) biacetyl (Exp III), and d) biacetyl (Exp IV). The mass spectra are shifted for the sake of clarity. The experimental conditions used are provided in Table 1.

In the absence of calibration standards for the SOZ molecules, quantum chemistry calculations were performed to find their binding energies with NH_4_^+^ ions and evaluate the instrument sensitivity. The SOZs obtained from the scavenging reaction of biacetyl with the *syn*-MVKOO and *anti*-MACROO isomers of C_4_ sCI, labeled as C_4_ sCI-I SOZ and C_4_ sCI-II SOZ in Figure 3, were used for the binding energy calculations. Both of these isomers have slow rates of unimolecular isomerization^31,32^ and, thus, were expected to have relatively high mixing ratios in the chamber compared with the *anti*-MVKOO and *syn*-MACROO isomers. A total of 23 unique structures were identified, and the minimum energy structures of the SOZ complexes with NH_4_^+^ feature interactions between the NH_4_^+^ ion, the carbonyl oxygen of α-diketones, and the peroxide oxygens of the SOZ as shown in Figure 3. Higher-energy structural conformations exhibit interactions between NH_4_^+^, the carbonyl oxygen of α-diketones, and the ether oxygen of the SOZ. The relative energies of various structures for the ozonide and the NH_4_^+^ complexes are provided in Tables S3–S8 in the Supporting Information. The lowest-energy conformers of SOZ and the NH_4_^+^ complex were used to calculate the binding energy value shown in Figure 3. Binding energies estimated at the drift tube temperature of 333 K using the Boltzmann factor weighted average energies for the secondary ozonides and the NH_4_^+^ complex were found to be within 1% of the values obtained using the minimum energy conformers. The calculated binding energies for the C_1_ and C_4_ sCI SOZs complexes are high ∼ 150 kJ mol^–1^ and within 10%. The SOZs derived from the scavenging reaction of acetyl propionyl were expected to have similar binding energies. These calculations and observations confirm that the ion signals at *m*/*z* 150, 164 and 190, 204 result from the C_1_ sCI and C_4_ sCI SOZs complexed with NH_4_^+^ and the signal strengths should be comparable for quantitative kinetic analysis. The C_1_ sCI SOZ (*m*/*z* 150/164) and C_4_ sCI SOZ (*m*/*z* 190/204) labels were used to represent SOZs from both scavengers in the analysis shown in the next section.

**Figure 3:**
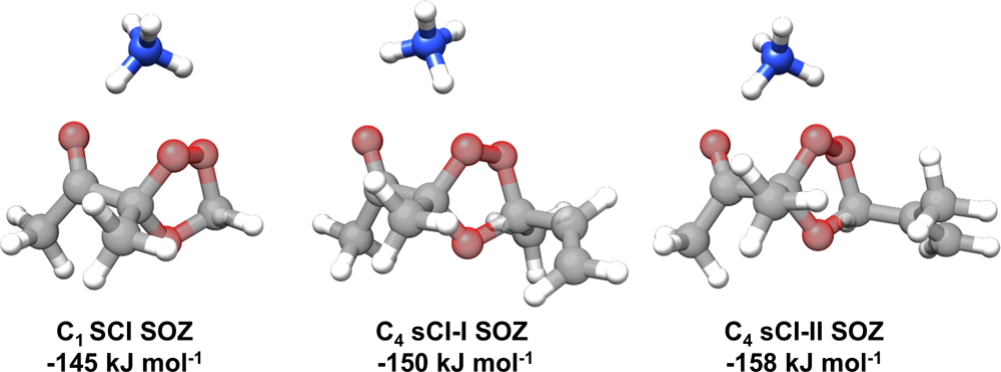
Minimum energy structures for the complexes of secondary ozonides with NH_4_^+^ obtained using the biacetyl scavenger and the corresponding binding energies calculated at the B3LYP-D3BJ/6–311++G­(d,p) level of theory. C_4_ sCI-I and C_4_ sCI-II represent *syn*-MVKOO and *anti*-MACROO structures as shown in Figure 1. The carbon and oxygen atoms in red and gray colors are made semitransparent for clarity.

### 3.2 Relative Yield of C_1_ and C_4_ Stabilized Criegee Intermediates

Figure 4a-c shows the temporal profiles for the various sCI SOZ masses observed in Exp II–IV. First, an empirical kinetic model was used to estimate the relative yields of the C_1_ and C_4_ sCIs. This simplified model assumes that the isoprene ozone reaction produces only C_1_ sCI and C_4_ sCIs, [Disp-formula eqR1] and [Disp-formula eqR2], and the SOZ traces were used in the fits to find the relative yields. The mass spectrometric measurements cannot distinguish between the various isomers of C_4_ SCI SOZs; therefore, this analysis assumed contributions from all isomers to the C_4_ sCI SOZ mass signals. The SOZ signals were reproduced well by using a *pseudo*-first-order loss process for isoprene. The ozone concentrations used during the experiments were always in excess. The sCIs were expected to react rapidly with α-diketones over millisecond time scales forming SOZ and were also assumed to be under pseudo-first-order conditions, [Disp-formula eqR3] and [Disp-formula eqR4]. The SOZs then cluster with NH_4_^+^ ions upon entering the drift tube. The clustering process and the ion transmission rates were expected to be similar for both SCIs and thus not modeled explicitly.^40^ Both SOZ signals show losses over longer time scales, consistent with previous observations, and a first-order loss process, [Disp-formula eqR5] and [Disp-formula eqR6], was also included in the model to account for this.^35,41^ The rate coefficients for reactions [Disp-formula eqR1], [Disp-formula eqR2], [Disp-formula eqR5], and [Disp-formula eqR6] and the initial isoprene signal were varied in a numerical fitting model using the odeint function of the SciPy module for solving ordinary differential equations and the minimize function of the LMFIT module in Python.

**Figure 4:**
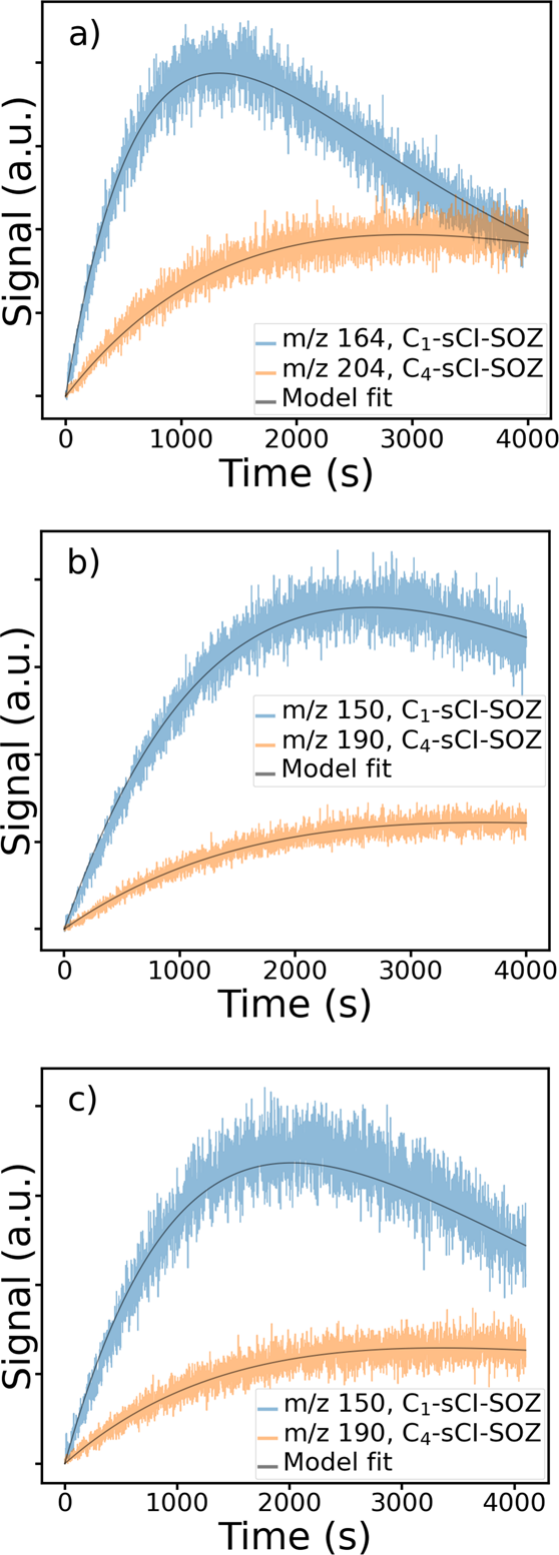
Temporal traces for the selected *m*/*z* signals from a) acetylpropionyl (Exp II), b) biacetyl (Exp III), and c) biacetyl (Exp IV) experiments shown in Figure 2. Fit traces obtained using the empirical model discussed in the text are also shown.


R1 $$C_{5}H_{8}\left(+O_{3}\right)\to C_{1}\text{‐}\mathrm{sCI}$$

R2 $$C_{5}H_{8}\left(+O_{3}\right)\to C_{4}\text{‐}\mathrm{sCI}$$

R3 $$C_{1}\text{‐}\mathrm{sCI}\left(+\alpha \text{‐}\mathrm{diketone}\right)\to C_{1}\text{‐}\mathrm{sCI}\text{‐}\mathrm{SOZ}$$

R4 $$C_{4}\text{‐}\mathrm{sCI}\left(+\alpha \text{‐}\mathrm{diketone}\right)\to C_{4}\text{‐}\mathrm{sCI}\text{‐}\mathrm{SOZ}$$

R5 $$C_{1}\text{‐}\mathrm{sCI}\text{‐}\mathrm{SOZ}\to \mathrm{Product}1$$

R6 $$C_{4}\text{‐}\mathrm{sCI}\text{‐}\mathrm{SOZ}\to \mathrm{Product}2$$

The quality of the fit is good for all the experimental traces, as shown in Figure 4, and the relative yield values for the C_4_ sCI channel are provided in Table 2. The rate coefficient for scavenging reaction [Disp-formula eqR3] was fixed to values estimated using the concentration used and the bimolecular rate coefficient value reported by Murray and co-workers.^33^ The rate coefficient for [Disp-formula eqR4] was fixed to the same value as in [Disp-formula eqR3]. Fits performed using the constraints, *k*_4_ = 10 × *k*_3_ or *k*_4_ = *k*_3_/10, did not show significant differences in the fit result. Fit parameters were highly correlated for Exp III and IV; however, Exp II fit parameters had low correlations (shown in Table S1 in Supporting Information) which ensured good determination of the relative yield value. The fast decay of the C_1_-sCI-SOZ in Exp II likely provides good kinetic separation of the rise and the decay events and thus lowers the correlation between the rise constants for SOZ signals. The production and loss rates for SOZ signals in Exp III and IV were expected to be similar. Although these values are within 1σ, Exp III values are consistently smaller than Exp IV likely because of lower chamber temperature. Exp III and Exp IV were performed in the months of April and June, respectively, and the chamber temperatures were around 290 and 294 K. The relative yield value of C_4_ sCI is consistent for all experiments, as shown in the last column of Table 2, which suggests efficient scavenging and derivatization of the sCIs. The three C_4_ sCI relative yield values were averaged to provide the best estimated value of 0.19 ± 0.07.

**Table 2:** Fitted Parameters Were Obtained Using the Empirical Model ([Disp-formula eqR1]–[Disp-formula eqR6])­^a^

**Exp**	**k**_**1**_ **(10** ^**‑4**^ **s** ^**‑1**^ **)**	**k**_**2**_ **(10** ^**‑5**^ **s** ^**‑1**^ **)**	**k**_**3**_ **(s** ^**‑1**^ **)**	**k**_**5**_ **(10** ^**‑4**^ **s** ^**‑1**^ **)**	**k**_**6**_ **(10** ^**‑4**^ **s** ^**‑1**^ **)**	**k**_**2**_ **/(k**_**1**_ **+k**_**2**_ **)**
II	3.74 ± 0.01	8.22 ± 0.04	1290	11.39 ± 0.05	2.39 ± 0.03	0.180 ± 0.001
III	3.0 ± 0.8	7.5 ± 2.0	1200	3.8 ± 1.0	1.9 ± 0.7	0.20 ± 0.06
IV	4.0 ± 0.6	9.8 ± 1.5	595	4.9 ± 0.7	1.6 ± 0.4	0.20 ± 0.03

a The *k*_3_ and *k*_4_ values were assumed to be the same and were fixed in the fit. The fit traces are shown in Figure 4. Detail of the fitting procedure is provided in the main text and Table S1 in the Supporting Information.

### 3.3 Isomer Specific Yields of C_4_ Stabilized Criegee Intermediates

An explicit model, [Disp-formula eqR7]-[Disp-formula eqR37], was used to find the yields of the various isomers of C_4_ sCI. The SOZ signals were calibrated, using a scaling factor, with respect to isoprene signal using the directly measured C_1_ sCI yield value of 0.6 by Nguyen et al.^9^ The model accounts for production of C_1_ sCI through isoprene ozonolysis and also through ozonolysis of methyl vinyl ketone and methacrolein, which are produced from the initial ozonolysis. The ozonolysis rate coefficients were taken from the IUPAC recommendations.^8^ The prompt and thermalized formation of OH radical from ozonolysis of isoprene, methyl vinyl ketone, and methacrolein is also included. This is important as OH scavenging by cyclohexane can produce HO_2_ which reacts with O_3_, [Disp-formula eqR31]-[Disp-formula eqR37]. Computational studies by Zhang and Zhang suggested a 50/50 split between prompt vs thermal OH yields, and Kuwata et al. suggested a prompt yield of 0.14 from isoprene ozonolysis.^29,42^ The total OH yield is reported at ∼ 0.28 from various experimental studies under ambient conditions.^7^ Thus, a prompt OH yield value of 0.14 was used in the model. The C_4_ sCI yields were divided into slow (*syn-*MVKOO, *anti-*MACROO) and fast (*anti-*MVKOO, *syn-*MVKOO) isomerizing channels. The slow isomerizing channels were expected to be the primary contributor to the relative yield estimated in the last section and were constrained to a yield of 0.11. This results in a yield of 0.15 for the fast-isomerizing channels. The thermalized unimolecular reaction rates of C_4_ sCIs were obtained from a combination of direct measurements or high-level quantum chemistry calculations.^31,32,43^ Reactions of Criegee intermediates with ozone and secondary products such as formaldehyde were not expected to compete with the scavenging reactions of α-diketones based on reported rate coefficients and were not included in the model.^44,45^ All of the reactions and rate coefficients used in the fit model are shown below. The units for unimolecular and bimolecular reaction rate coefficients are s^–1^ and cm^3^ molecule^–1^ s^–1^, respectively. Initial concentrations used are listed in Table 1 for the various reactants.

Nguyen et al.^9^:

R7 $$\begin{array}{c} C_{5}H_{8}+O_{3}\to {\mathrm{CH}}_{2}\mathrm{OO}+\mathrm{MVK}\\ k_{7}=0.42\times 1.27\times 10^{-17} \end{array}$$

Nguyen et al.^9^:

R8 $$\begin{array}{c} C_{5}H_{8}+O_{3}\to {\mathrm{CH}}_{2}\mathrm{OO}+\mathrm{MACR}\\ k_{8}=0.18\times 1.27\times 10^{-17} \end{array}$$

See text:

R9 $$\begin{array}{c} C_{5}H_{8}+O_{3}\to {\mathrm{CH}}_{2}O+\mathrm{OH}\\ k_{9}=0.14\times 1.27\times 10^{-17} \end{array}$$

R10 $$\begin{array}{c} C_{5}H_{8}+O_{3}\to \mathrm{syn}\text{‐}\mathrm{MVKOO}+{\mathrm{CH}}_{2}O\\ k_{10}=a\left(\mathrm{varied}\right)\times 1.27\times 10^{-17} \end{array}$$

R11 $$\begin{array}{c} C_{5}H_{8}+O_{3}\to \mathrm{anti}\text{‐}\mathrm{MVKOO}+{\mathrm{CH}}_{2}O\\ k_{11}=b\left(\mathrm{varied}\right)\times 1.27\times 10^{-17} \end{array}$$

R12 $$\begin{array}{c} C_{5}H_{8}+O_{3}\to \mathrm{syn}\text{‐}\mathrm{MACROO}+{\mathrm{CH}}_{2}O\\ k_{12}=c\times 1.27\times 10^{-17}\left[c=0.15-b\right] \end{array}$$

R13 $$\begin{array}{c} C_{5}H_{8}+O_{3}\to \mathrm{anti}\text{‐}\mathrm{MACROO}+{\mathrm{CH}}_{2}O\\ k_{13}=d\times 1.27\times 10^{-17}\left[d=0.11-a\right] \end{array}$$

IUPAC^8^:

R14 $$\begin{array}{c} \mathrm{MVK}+O_{3}\to {\mathrm{CH}}_{2}\mathrm{OO}+\mathrm{product}\\ k_{14}=0.84\times 5.2\times 10^{-18} \end{array}$$

IUPAC^8^:

R15 $$\begin{array}{c} \mathrm{MVK}+O_{3}\to \mathrm{OH}+\mathrm{other products}\\ k_{15}=0.16\times 5.2\times 10^{-18} \end{array}$$

IUPAC^8^:

R16 $$\begin{array}{c} \mathrm{MACR}+O_{3}\to {\mathrm{CH}}_{2}\mathrm{OO}\\ k_{16}=0.58\times 1.2\times 10^{-18} \end{array}$$

IUPAC^8^:

R17 $$\begin{array}{c} \mathrm{MACR}+O_{3}\to \mathrm{OH}+\mathrm{other products}\\ k_{17}=0.2\times 1.2\times 10^{-18} \end{array}$$

IUPAC^8^:

R18 $$\begin{array}{c} \mathrm{MACR}+O_{3}\to \mathrm{other products}\\ k_{18}=0.22\times 1.2\times 10^{-18} \end{array}$$

Barber et al.^31^:

R19 $$\begin{array}{c} syn\text{‐}\mathrm{MVKOO}\to \mathrm{OH}+\mathrm{product}\\ k_{19}=33 \end{array}$$

Barber et al.^31^:

R20 $$\begin{array}{c} anti\text{‐}\mathrm{MVKOO}\to \mathrm{dioxole}\\ k_{20}=2140 \end{array}$$

Lin et al.^32^:

R21 $$\begin{array}{c} syn\text{‐}\mathrm{MACROO}\to \mathrm{dioxole}\\ k_{21}=2600 \end{array}$$

Lin et al.^32^:

R22 $$\begin{array}{c} anti\text{‐}\mathrm{MACROO}\to \mathrm{dioxirane}\\ k_{22}=7 \end{array}$$

Stone et al.^46^:

R23 $$\begin{array}{c} {\mathrm{CH}}_{2}\mathrm{OO}\to \mathrm{dioxirane}\\ k_{23}=0.2 \end{array}$$

Cornwell et al.^33^:

R24 $$\begin{array}{c} {\mathrm{CH}}_{2}\mathrm{OO}+\mathrm{\alpha ‐diketone}\to C_{1}\text{‐}\mathrm{sCI SOZ}\\ k_{24}=1.45\times 10^{-11},\left(\mathrm{biacetyl}\right)\\ k_{24}=1.29\times 10^{-11},\left(\mathrm{acetylpropionyl}\right) \end{array}$$

Cornwell et al.^33^:

R25 $$\begin{array}{c} syn\text{‐}\mathrm{MVKOO}+\mathrm{\alpha ‐diketone}\to C_{4}\text{‐}\mathrm{sCI SOZ}\\ k_{25}=k_{24} \end{array}$$

Cornwell et al.^33^:

R26 $$\begin{array}{c} anti\text{‐}\mathrm{MVKOO}+\mathrm{\alpha ‐diketone}\to C_{4}\text{‐}\mathrm{sCI SOZ}\\ k_{26}=k_{24} \end{array}$$

Cornwell et al.^33^:

R27 $$\begin{array}{c} syn\text{‐}\mathrm{MACROO}+\mathrm{\alpha ‐diketone}\to C_{4}\text{‐}\mathrm{sCI SOZ}\\ k_{27}=k_{24} \end{array}$$

Cornwell et al.^33^:

R28 $$\begin{array}{c} anti\text{‐}\mathrm{MACROO}+\mathrm{\alpha ‐diketone}\to C_{4}\text{‐}\mathrm{sCI SOZ}\\ k_{28}=k_{24} \end{array}$$

R29 $$\begin{array}{c} C_{1}\mathrm{sCI ozonide}\to \mathrm{products}\\ k_{29}=\mathrm{varied} \end{array}$$

R30 $$\begin{array}{c} C_{4}\mathrm{sCI ozonide}\to \mathrm{products}\\ k_{30}=\mathrm{varied} \end{array}$$

Atkinson et al.^47^:

R31 $$\begin{array}{c} \mathrm{OH}+C_{6}H_{12}\to C_{6}H_{11}+H_{2}O\\ k_{31}=7.0\times 10^{-12} \end{array}$$

Platz et al.^48^:

R32 $$\begin{array}{c} C_{6}H_{11}+O_{2}\to {\mathrm{RO}}_{2}\\ k_{32}=1.3\times 10^{-11} \end{array}$$

Rowley et al.^49^:

R33 $$\begin{array}{c} {\mathrm{RO}}_{2}+{\mathrm{RO}}_{2}\to \mathrm{RO}+\mathrm{RO}+O_{2}\\ k_{33}=2.8\times 10^{-14} \end{array}$$

Orlando et al.^50^:

R34 $$\begin{array}{c} \mathrm{RO}+O_{2}\to {\mathrm{HO}}_{2}+\mathrm{product}\\ k_{34}=7.6\times 10^{-15} \end{array}$$

Rowley et al.^51^:

R35 $$\begin{array}{c} {\mathrm{RO}}_{2}+{\mathrm{HO}}_{2}\to \mathrm{ROOH}+O_{2}\\ k_{35}=1.8\times 10^{-11} \end{array}$$

IUPAC^8^:

R36 $$\begin{array}{c} {\mathrm{HO}}_{2}+{\mathrm{HO}}_{2}\to H_{2}O_{2}+O_{2}\\ k_{36}=1.6\times 10^{-12} \end{array}$$

IUPAC^8^:

R37 $$\begin{array}{c} {\mathrm{HO}}_{2}+O_{3}\to \mathrm{OH}+2O_{2}\\ k_{37}=2\times 10^{-15} \end{array}$$

Figure 5 shows the fitted traces obtained using the explicit model for Exp II–IV. There are some small deviations at longer time scales for isoprene likely because of overestimated O_3_ concentration in the model or kinetic interference from ozonolysis products, particularly from C_4_ sCI unimolecular reactions. The C_1_ sCI SOZ produced during the acetylpropionyl experiments has a decay rate significantly faster than that of the biacetyl experiment. Previously, C_1_ sCI SOZs from various carbonyl compounds have been predicted to decompose to formic acid or ester.^52^ The acetyl propionyl experiment showed an increase in a signal consistent with the ester and NH_4_^+^ complex mass, which was absent in the biacetyl experiment. The temporal profile of this signal at *m*/*z* 134 is shown in Figure S4 in the Supporting Information, and rise rate is consistent with the decay rate of the C_1_ sCI SOZ signal. The SOZ decay rate coefficient values, *k*_29_ and *k*_30_, are around a factor of 2 lower than *k*_5_ and *k*_6_ values obtained from the empirical model. This is likely because of the explicit modeling of the scavenging reaction compared with the pseudo first order approximation in the empirical model. The fitted parameters and their correlations from the explicit model fits are provided in Tables 3 and S2 in the Supporting Information. The fit errors were small, < 1% and, thus, are not shown in Table 3. The correlations between the C_4_ sCI yield values (a, b) were −0.6, −0.9, and −0.3 for Exp II, III, and IV. The fitted yield values between various experiments are within 10% as shown in Table 3. We expect systematic error of at least 20% derived from the fitted scaling factor for the SOZ signals from experiments III and IV which should be same. This likely arises from a combination of variations in the chemical and physical conditions in the chamber or during the sampling and ionization processes. Possible systematic errors from the model are discussed in the next section. Scaling of 5–7 for the SOZ signal would be consistent with a clustering ionization mechanism compared with the proton transfer mechanism for isoprene ionization in a low-pressure and high-temperature drift tube. Overall, all fits consistently showed a lower yield for the MVKOO isomers compared with the MACROO isomers and a similar yield for *syn*-MVKOO and *anti*-MACROO.

**Figure 5:**
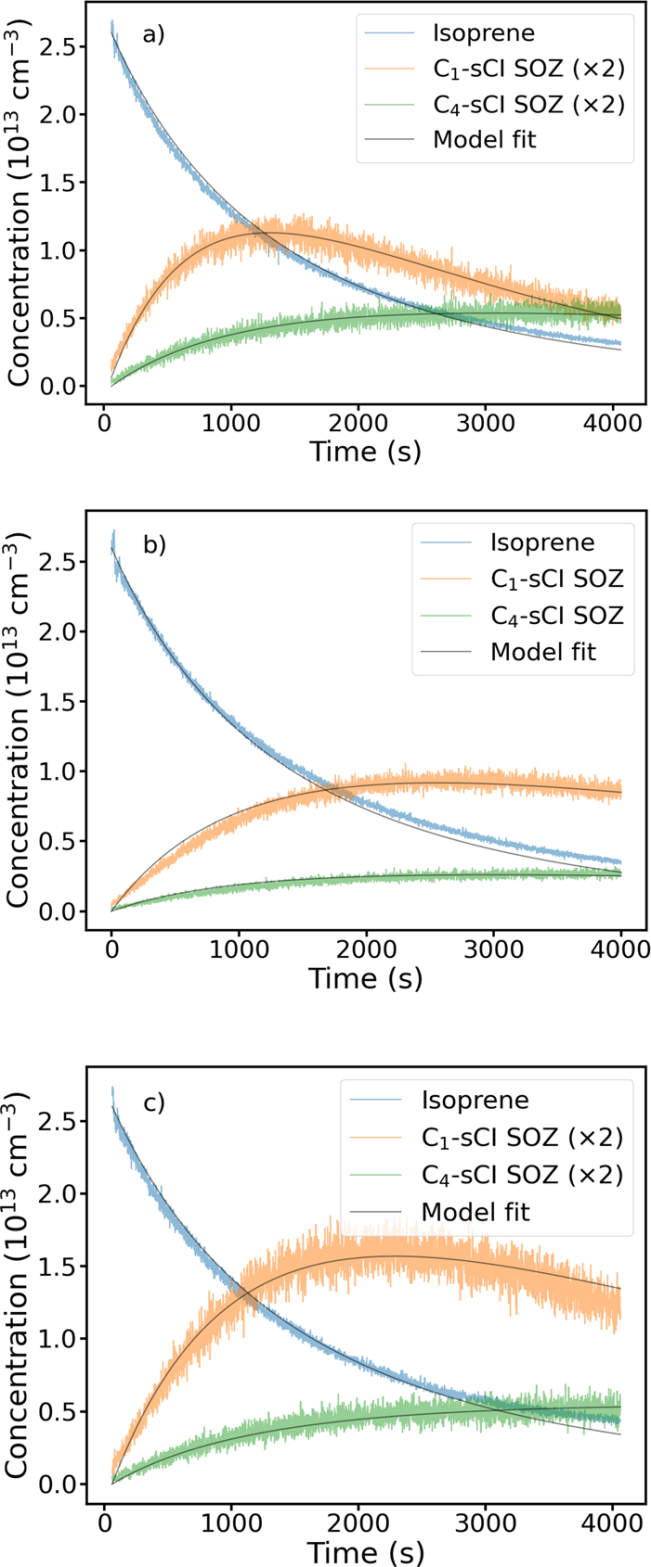
Explicit model fit for the a) acetylpropionyl (Exp II), b) biacetyl (Exp III), and c) biacetyl (Exp IV) experiments. Initial concentrations are provided in Table 1, and details of the model fit are provided in the main text. The SOZ traces in a) and c) have been scaled by a factor of 2 for clarity.

**Table 3:** Fitted Parameters Were Obtained Using the Explicit Model ([Disp-formula eqR7]-[Disp-formula eqR37])­^a^

**Exp**	**k**_**10**_ **/k**_**O3**_	**k**_**11**_ **/k**_**O3**_	**k**_**12**_ **/k**_**O3**_	**k**_**13**_ **/k**_**O3**_	**k**_**29**_ **(10** ^**‑4**^ **s** ^**‑1**^ **)**	**k**_**30**_ **(10** ^**‑4**^ **s** ^**‑1**^ **)**	**Scaling**
Exp II	0.046	0.048	0.102	0.064	7.06	1.19	7.44
Exp III	0.057	0.050	0.100	0.053	1.80	1.21	5.67
Exp IV	0.049	0.053	0.097	0.061	2.61	0.37	4.95

a The fit traces are listed in Figure 5. The IUPAC recommended total ozonolysis rate coefficient value, k_O3_, of 1.27 × 10^–17^ cm^3^ molecule^–1^ s^–1^ was used in the fit.^8^ Details of the fitting procedure are provided in the main text and Table S2 in the Supporting Information.

## 4 Discussion

The *k*_2_/(*k*_1_ + *k*_2_) value of 0.19 ± 0.07 obtained from the empirical model fit provides a good estimate for the relative yield of the C_4_ sCI. This value is in good agreement with the previous study of Sipila et al., who suggested ∼ 15% contribution of C_4_ sCIs to the total sCI yield from isoprene ozonolysis.^17^ A chamber study by Rickard et al. showed increased yields of methyl vinyl ketone and methacrolein by 15 to 25% upon addition of SO_2_ during isoprene ozonolysis.^23^ This suggests conversion of SO_2_ to SO_3_ along with the carbonyl products and thus a significant yield of C_4_ SCIs.^28^ Computational studies consistently predict significant yields of C_4_ sCIs.^29,30,42^ Each of these observations and predictions contrast with the conclusion of the Nguyen et al. study which suggested a low yield of C_4_ sCIs.^9^ This study measures the derivatized Criegee intermediate mass directly and thus provides yield values with a higher level of certainty.

The C_4_ sCIs are expected to have fast and slow isomerizing pathways which were included in the explicit model.^31,32^ Under the conditions used during the experiments, only the slow isomerizing C_4_ sCI structures, *syn*-MVKOO and *anti*-MACROO, were expected to survive long enough for scavenging to occur. However, there may be some contribution from the fast isomerizing, *anti*-MVKOO and *syn*-MACROO, in the C_4_ sCI relative yield value obtained from the empirical model fit. Experiments with higher concentrations of α-diketones to increase the scavenging rate and characterize the fast-isomerizing structures were not performed as the diketones react with NH_4_^+^ and can significantly interfere with the ion chemistry inside the drift tube. A suitable scavenger that can measure C_4_ sCI yields over a large range of scavenging rates could probe both fast and slow isomerizing C_4_ sCI structures. Using the total sCI yield of 0.6 ± 0.1 reported by Wennberg and co-workers, a combined C_4_ sCI yield value of 0.11 ± 0.05 is estimated for *syn*-MVKOO and *anti*-MACROO structures.^7^ The estimated error accounts for any systematic errors from the measurements as described in section 3.3 and any interference from the fast isomerizing *anti*-MVKOO and *syn*-MACROO structures during the scavenging process.

Explicit model fitting performed to find the contribution from various isomers of C_4_ sCI is dependent on the C_1_ sCI and the prompt OH yield from isoprene ozonolysis under ambient conditions. The C_1_ sCI yield value has now been confirmed by various methods,^7^ but the prompt OH yield under ambient condition remains uncertain. The total OH yield from the model fit obtained by adding the assumed prompt yield and yield of *syn*-MVKOO is 0.19, which is significantly lower than the previous measurements of ∼ 0.28. This suggests a higher prompt OH yield and a lower yield for fast-isomerizing C_4_ sCI structures compared to our model assumption. These parameters are highly correlated and thus cannot be determined from our model fit analysis. Further measurements are needed in the future to independently constrain these two channels and fully characterize the branching of the isoprene ozonolysis reaction.

## 5 Conclusions

A new method has been developed to measure the mass speciated yields of sCIs generated from the ozonolysis of BVOCs providing fresh insights into the well-studied isoprene ozonolysis reaction. In the absence of direct field measurements of Criegee intermediates, bottom-up modeling studies are heavily reliant on the kinetic parameters obtained from laboratory studies such as this. The mass speciated yield for C_4_ sCIs from this study provides further support for the presence of non-negligible concentrations of Criegee intermediates in the troposphere. These measurements support previous modeling studies, based on indirect estimates, that attribute a significant fraction of SO_2_ oxidation to Criegee intermediates, particularly in forested regions.^25,53^ In principle, this approach can be extended to obtain mass speciated yield measurements of the larger Criegee intermediates generated from terpenes, and we anticipate that future experiments of this type will provide better constraints for models and more accurate impact assessments of Criegee intermediate chemistry. Furthermore, a recent study reported accretion products of C_1_ sCI in the gas and condensed phases over the Amazon rainforest would seem to imply higher mixing ratios of Criegee intermediates than previously reckoned.^54^ Accretion products such as these, as with other highly oxygenated molecules, have low volatility and have been shown to initiate pure biogenic nucleation, which may be important for particle formation processes in pristine environments.^55^

## Supplementary Material



## Data Availability

Experimental kinetic and mass spectra data and outputs of the quantum chemistry calculations are archived in the University of Nottingham’s Research Data Storage Facility. DOI: 10.17639/nott.7592

## Notes

Figure S1 Ion yields as a function of reduced electric field for various *m*/*z* for Exp II; Figure S2 Ion yields as a function of reduced electric field for various *m*/*z* for Exp III; Figure S3 Full temporal scans from Exp II; Figure S4 Full temporal scans from Exp III; Figure S5 Background signal and subtraction at *m*/*z* 150 for Exp III, Figure S6 Comparison of signal at *m*/*z* 150 for Exp I and II; Table S1 Fit parameter obtained from the empirical fits; Table S2 Fit parameters obtained from explicit fits; Table S3 Relative energy of structures for C_1_ sCI SOZ NH_4_^+^ complex; Table S4 Relative energy of structures for C_1_ sCI SOZ; Table S5 Relative energy of structures for C_4_ sCI-I SOZ NH_4_^+^ complex; Table S6 Relative energy of structures for C_4_ sCI-II SOZ NH_4_^+^ complex; Table S7 Relative energy of structures for C_4_ sCI-I SOZ complex; Table S8 Relative energy of structures for C_4_ sCI-II SOZ complex (PDF)
