# Supplementary material for: Yield of the Four-Carbon Stabilized Criegee Intermediates from Isoprene Ozonolysis
Source: ACS Earth Space Chem. 2026 Jan 8;10(2):328–37. doi: 10.1021/acsearthspacechem.5c00226 (PMC12927010; doi:10.1021/acsearthspacechem.5c00226)
Supplement: Supplementary file 1 [file sp5c00226_si_001.pdf]

Yield of the Four-carbon Stabilized Criegee Intermediates from Isoprene Ozonolysis

Rabi Chhantyal-Pun,[1] Pengcheng Wang,[2] Shefali Baweja,[1] Joseph Bainbridge,[1] Chenyang Xue,[2] Véronique Daële,[2] Abdelwahid Mellouki,[3] Max R. McGillen[2]

[1]School of Chemistry, University of Nottingham, UK, [2]CNRS, Institut de Combustion Aérothermique Réactivité et Environnement (ICARE), Orléans, France, [3]Mohammed VI Polytechnic University, Ben Guerir 43150, Morocco

Corresponding Authors

Rabi Chhantyal-Pun, Email: r.chhantyalpun@nottingham.ac.uk

Max McGillen, Email: max.mcgillen@cnrs-orleans.fr

Supporting Information

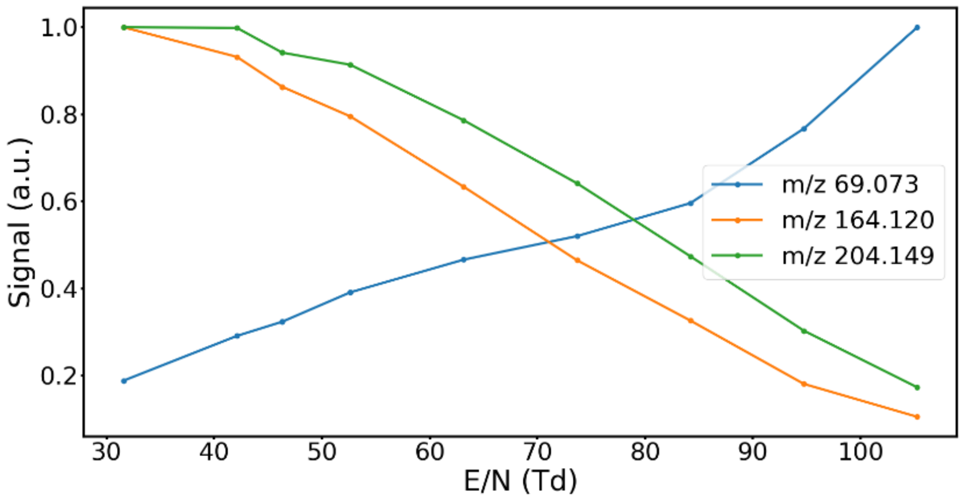

Figure S1: Yields of the various ions as a function of the drift tube electric field during the ozonolysis of isoprene in presence of acetylpropionyl (Exp II). All signals are corrected for the changes in concentrations over the scan duration and are scaled to their respective maximum values for clarity. The isoprene signal at m/z 69 shows an increase with the increase in the electric field strength which results from more efficient proton transfer at higher collision energies. The kinetic traces shown in Figures 4, 5, S3 and S4 were obtained at E/N value of 46.3 Td.

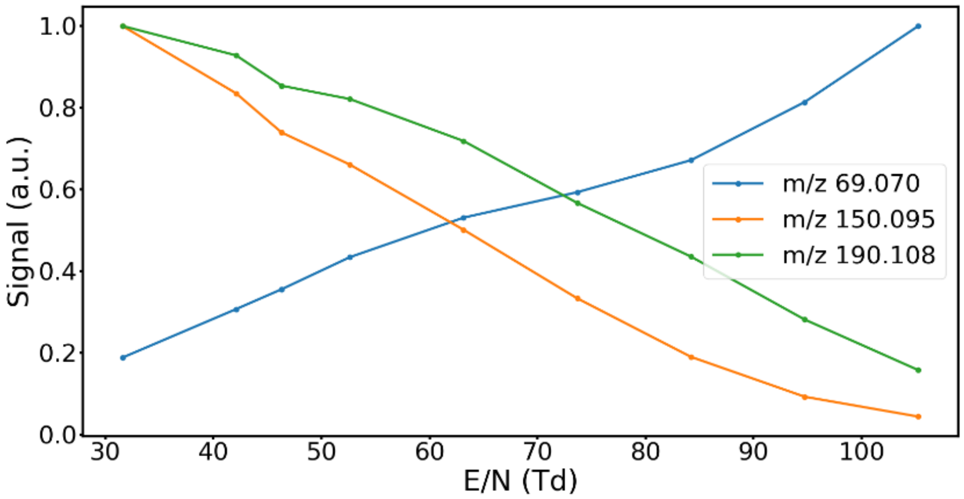

Figure S2: Yields of the various ions as a function of the drift tube electric field during the ozonolysis of isoprene in presence of biacetyl (Exp III). All signals are corrected for the changes in concentrations over the scan duration and are scaled to their respective maximum values for clarity. The m/z 150 signal has some interference as shown in Figure S4 and thus the rate of decrease may be enhanced. The kinetic traces shown in Figure 4, 5, S3 and S4 were obtained at lower E/N value of 46.3 Td.

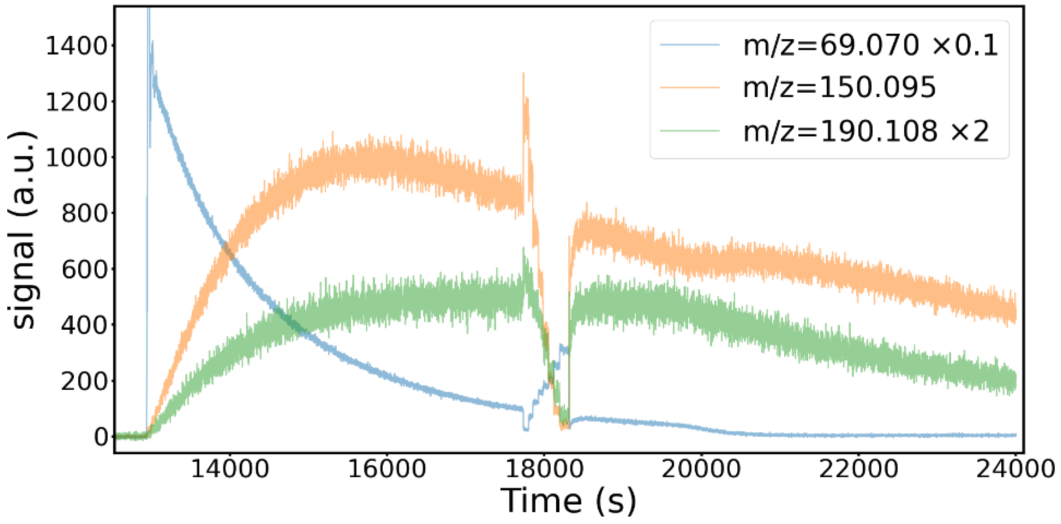

Figure S3. Full temporal profile of the various mass signals from the biacetyl experiment (Exp III). The isoprene signal at m/z 69 was reduced by a factor of 10 and the C_4 sCI ozonide signal at m/z 190 was increased by a factor of 2 for better comparison. Ozone concentration was increased to 2×10^{14} cm^{-3} at around 20000s to test the reactivity of the ozonides. The drop in signal around 18000s is because of the electric field strength measurements.

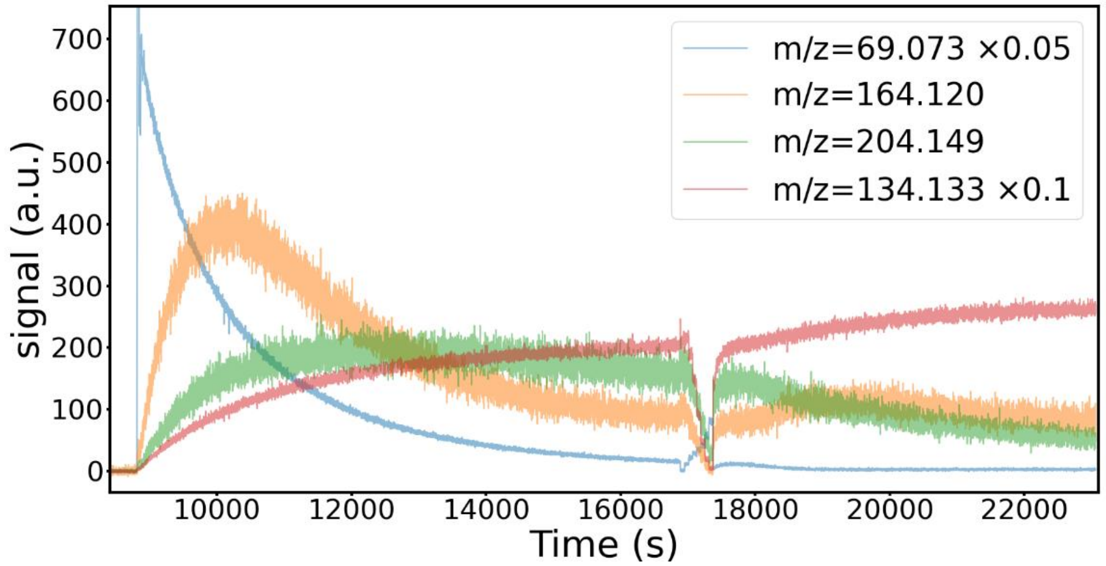

Figure S4. Full temporal profile of the various mass signals from the acetylpropionyl experiment (Exp II). The isoprene signal at m/z 69 and ester signal at 134 were reduced by a factor of 20 and 5 for better comparison. Ozone concentration was increased to 2×10^{14} cm^{-3} at around 19000s to test the reactivity of the ozonides. The drop in signal around 18000s is because of the electric field strength measurements the results of which are shown in Figure S1.

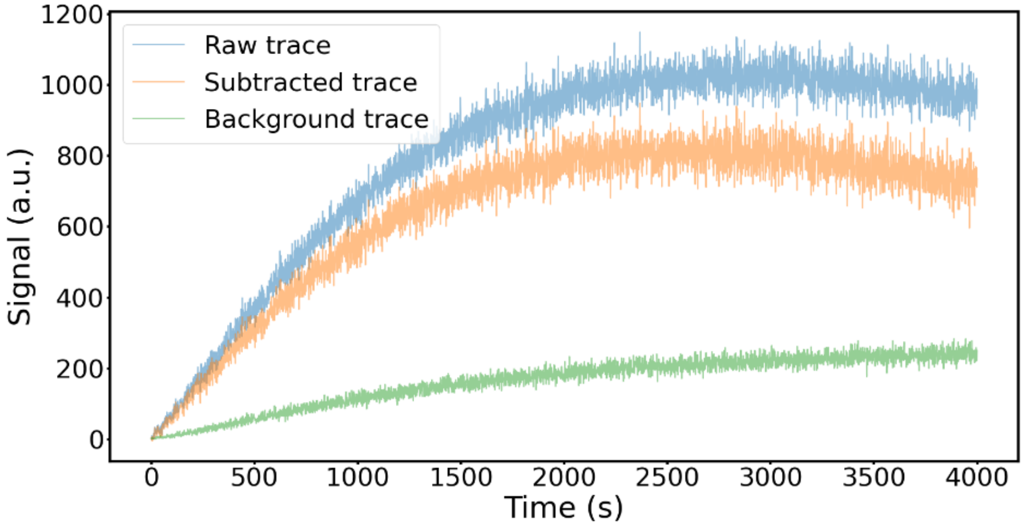

Figure S5. Background signal subtraction of the m/z 150 signal for the biacetyl traces shown in Figure 4 and 5 in the main text. The background trace was obtained from an ozonolysis experiment performed in absence of biacetyl as shown in Figure S6.

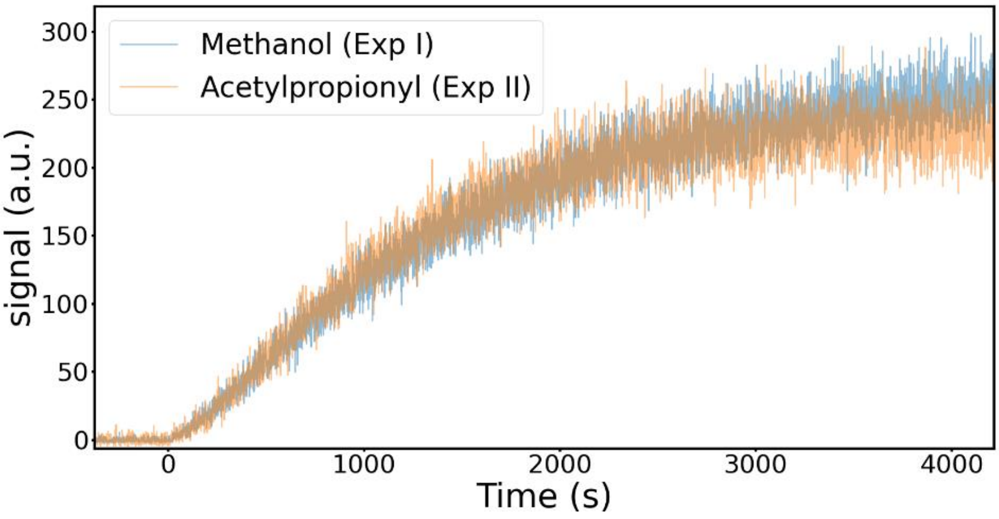

Figure S6. Background signal at m/z 150 obtained during isoprene ozonolysis experiments in presence of acetylpropionyl or methanol scavenger. The trace intensities were scaled with respect to the isoprene signal. The methanol trace (Exp I) was used for background subtraction as shown in Figure S5.

Table S1: Fitted parameters and their correlation obtained using the empirical model described in the main text. Unreported correlations are less than 0.1.

Exp	Fitted Parameters	Correlations
Exp II	A [Initial isoprene signal]: 2178 ± 4 (0.20%) (init = 1000)<br>k1: (3.77 ± 0.01) $\times 10^{-4}$ (0.28%) (init = 1×10^{-4})<br>k2: (0.831 ± 0.004) $\times 10^{-4}$ (0.50%) (init = 1×10^{-4})<br>k3: 1290 (fixed)<br>k4: 1290 (fixed)<br>k5: (11.32 ± 0.03) $\times 10^{-4}$ (0.31%) (init = 1×10^{-4})<br>k6: (2.37 ± 0.03) $\times 10^{-4}$ (1.29%) (init = 1×10^{-4})	C(k2, k6) = 0.742<br>C(A, k5) = 0.700<br>C(A, k1) = -0.452<br>C(k2, k5) = -0.434<br>C(A, k2) = -0.350<br>C(k1, k6) = -0.317<br>C(k1, k5) = 0.212<br>C(A, k6) = 0.210<br>C(k5, k6) = -0.171<br>C(k1, k2) = 0.151
Exp III	A: 2485 ± 639 (25.72%) (init = 1000)<br>k1: (3.0 ± 0.8) $\times 10^{-4}$ (25.74%) (init = 1×10^{-4})<br>k2: (0.7 ± 0.2) $\times 10^{-4}$ (27.08%) (init = 1×10^{-4})<br>k3: 1200 (fixed)<br>k4: 1200 (fixed)<br>k5: (3.8 ± 1.0) $\times 10^{-4}$ (26.08%) (init = 1×10^{-4})<br>k6: (1.9 ± 0.7) $\times 10^{-4}$ (38.07%) (init = 1×10^{-4})	C(A, k1) = -1.000<br>C(A, k5) = 1.000<br>C(k1, k5) = -1.000<br>C(k2, k5) = -1.000<br>C(A, k2) = -1.000<br>C(k1, k2) = 1.000<br>C(k1, k6) = -0.999<br>C(A, k6) = 0.999<br>C(k5, k6) = 0.998<br>C(k2, k6) = -0.997
Exp IV	A: 1132 ± 157 (13.88%) (init = 1000)<br>k1: (4.0 ± 0.6) $\times 10^{-4}$ (13.91%) (init = 1×10^{-4})<br>k2: (1.0 ± 0.2) $\times 10^{-4}$ (15.46%) (init = 1×10^{-4})<br>k3: 595 (fixed)<br>k4: 595 (fixed)<br>k5: (4.9 ± 0.7) $\times 10^{-4}$ (14.25%) (init = 1×10^{-4})<br>k6: (1.6 ± 0.4) $\times 10^{-4}$ (25.31%) (init = 1×10^{-4})	C(A, k1) = -1.000<br>C(A, k5) = 1.000<br>C(k1, k5) = -0.999<br>C(k2, k5) = -0.999<br>C(A, k2) = -0.999<br>C(k1, k2) = 0.999<br>C(k1, k6) = -0.995<br>C(A, k6) = 0.995<br>C(k5, k6) = 0.994<br>C(k2, k6) = -0.989

Table S2: Fitted parameters and their correlation obtained using the explicit model described in the main text. Unreported correlations are less than 0.1.

Exp	Fitted parameters	Correleations
Exp II	scaling: 7.45 ± 0.02 (0.21%) (init = 1)<br>k24: 1.29×10^{-11} (fixed)<br>k29: $(7.06 \pm 0.02) \times 10^{-4}$ (0.27%) (init = 2.6×10^{-4})<br>k30: $(1.192 \pm 0.005) \times 10^{-4}$ (0.44%) (init = 1.4×10^{-4})<br>C1SOZ [Initial signal]: $(4 \pm 20) \times 10^{10}$ (4.85%) (init = 10^{12})<br>a: 0.04646 ± 0.00005 (0.10%) (init = 0.05)<br>b: 0.04809 ± 0.00005 (0.10%) (init = 0.05)<br>c: 0.10191 ± 0.00005 (0.05%) == '0.26-0.11-b'<br>d: 0.06354 ± 0.00005 (0.08%) == '0.11-a'	C(k30, b) = -0.898<br>C(scaling, k29) = -0.715<br>C(a, b) = 0.591<br>C(k30, a) = -0.575<br>C(scaling, C1SOZ) = 0.510<br>C(scaling, k30) = -0.376<br>C(k29, k30) = 0.365<br>C(k29, b) = -0.315<br>C(scaling, a) = 0.280<br>C(scaling, b) = 0.256<br>C(C1SOZ, a) = 0.165<br>C(C1SOZ, b) = 0.163<br>C(k30, C1SOZ) = -0.154<br>C(k29, C1SOZ) = -0.110
Exp III	scaling: 5.686 ± 0.006 (0.10%) (init = 1)<br>k24: 1.45×10^{-11} (fixed)<br>k29: $(1.778 \pm 0.003) \times 10^{-4}$ (0.15%) (init = 2.6×10^{-4})<br>k30: $(1.21 \pm 0.02) \times 10^{-4}$ (1.49%) (init = 1.4×10^{-4})<br>C1SOZ: 0 (fixed)<br>a: 0.0568 ± 0.0001 (0.17%) (init = 0.05)<br>b: 0.0495 ± 0.0001 (0.17%) (init = 0.05)<br>c: 0.1005 ± 0.0001 (0.09%) == '0.26-0.11-b'<br>d: 0.0532 ± 0.0001 (0.18%) == '0.11-a'	C(a, b) = -0.934<br>C(k29, b) = 0.336<br>C(scaling, b) = -0.322<br>C(scaling, k29) = -0.289<br>C(scaling, k30) = -0.238<br>C(k29, a) = -0.188<br>C(scaling, a) = 0.147<br>C(k29, k30) = 0.116
Exp IV	scaling: 4.947 ± 0.008 (0.17%) (init = 1)<br>k24: 1.45×10^{-11} (fixed)<br>k29: $(2.612 \pm 0.009) \times 10^{-4}$ (0.36%) (init = 2.6×10^{-4})<br>k30: $(3.7 \pm 0.2) \times 10^{-5}$ (5.19%) (init = 1.4×10^{-4})<br>C1SOZ: 0 (fixed)<br>a: 0.04927 ± 0.00003 (0.06%) (init = 0.05)<br>b: 0.05270 ± 0.00008 (0.15%) (init = 0.05)<br>c: 0.09729 ± 0.00008 (0.08%) == '0.26-0.11-b'<br>d: 0.06073 ± 0.00003 (0.05%) == '0.11-a'	C(scaling, k29) = -0.750<br>C(k29, b) = -0.533<br>C(scaling, b) = 0.443<br>C(scaling, k30) = -0.415<br>C(k29, k30) = 0.350<br>C(a, b) = -0.316<br>C(scaling, a) = 0.259<br>C(k30, b) = -0.170

Table S3. Relative energies with ZPE correction for C_1 sCI SOZ NH_4^+ complexes calculated at B3LYP-D3BJ/6-311++G(d,p) level of theory**.** The CH_2OO sCI and biacetyl were used to generate the SOZ structures.

**Conformer**	**Structure**	**Relative Energy (kJ mol^{-1})**
a	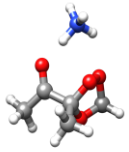	0
b	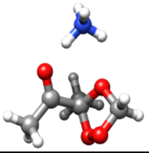	14.6
c	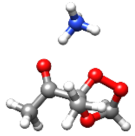	18.0
d	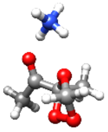	24.4

Table S4. Relative energies with ZPE correction for C_1 sCI SOZ calculated at B3LYP-D3BJ/6-311++G(d,p) level of theory**.** The CH_2OO sCI and biacetyl were used to generate the SOZ structures.

**Conformer**	**Structure**	**Relative Energy (kJ mol^{-1})**
a	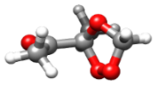	0
b	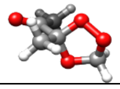	1.90
c	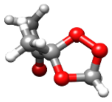	11.8

Table S5. Relative energies with ZPE correction for C_4 sCI-I SOZ NH_4^+ complexes calculated at B3LYP-D3BJ/6-311++G(d,p) level of theory**.** The *syn*-MVKOO isomer of C_4 sCI and biacetyl were used to generate the SOZ structures.

Conformer	Structure	Relative Energy (kJ mol^{-1})
a	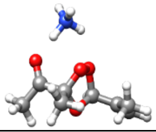	0.0
c	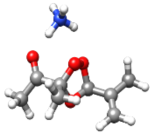	2.30
d	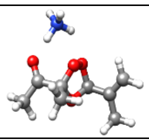	5.00
e	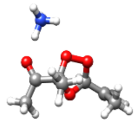	13.4
b	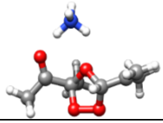	16.0
f	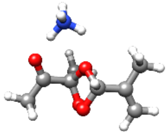	19.5
g	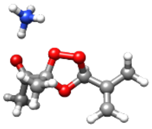	19.9
h	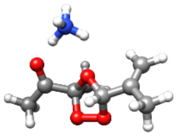	22.2
i	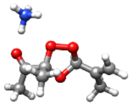	23.6

Table S6. Relative energies with ZPE correction for C_4 sCI-II SOZ NH_4^+ complexes calculated at B3LYP-D3BJ/6-311++G(d,p) level of theory. The *anti*-MACROO isomer of C_4 sCI and biacetyl were used to generate the SOZ structures.

Conformer	Structure	Relative Energy (kJ mol^{-1})
a	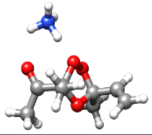	0.0
b	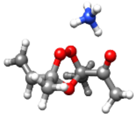	2.81
c	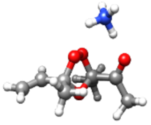	3.32
d	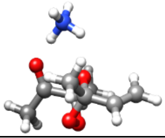	16.0
e	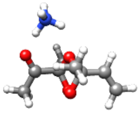	16.5
f	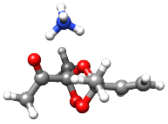	17.3
g	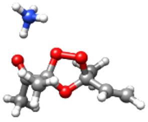	22.1
h	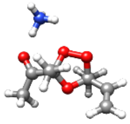	22.8
i	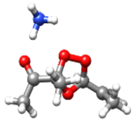	27.3
j	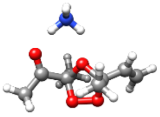	30.5

Table S7. Relative energies with ZPE correction for C_4 sCI-I SOZ calculated at B3LYP-D3BJ/6-311++G(d,p) level of theory**.** The *syn*-MVKOO isomer of C_4 sCI and biacetyl were used to generate the SOZ structures.

Conformer	Structure	Relative Energy (kJ mol^{-1})
a	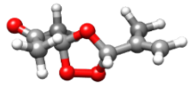	0
b	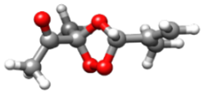	3.00
c	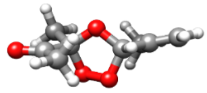	4.60
d	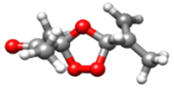	4.65
e	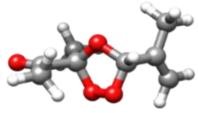	5.77
f	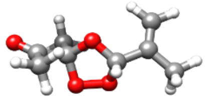	5.59
g	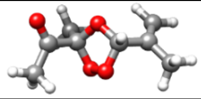	6.98
h	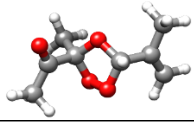	8.97
i	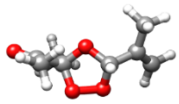	9.31
j	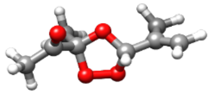	11.2
k	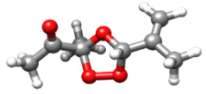	16.3
l	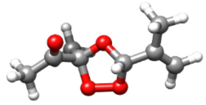	18.8

Table S8. Relative energies with ZPE correction for C_4 sCI-II SOZ calculated at B3LYP-D3BJ/6-311++G(d,p) level of theory**.** The *anti*-MACROO isomer of C_4 sCI and biacetyl were used to generate the SOZ structures.

**Conformer**	**Structure**	**Relative Eenergy (kJ mol^{-1})**
a	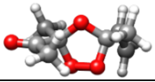	0
b	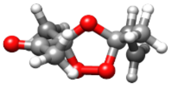	0.19
c	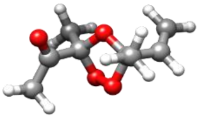	0.61
d	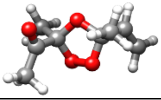	2.34
e	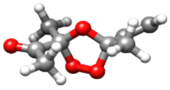	3.75
f	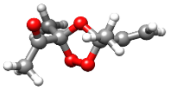	3.28
g	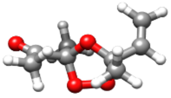	4.89
h	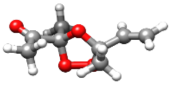	6.00
i	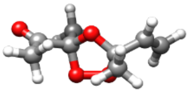	8.93
j	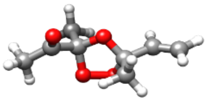	15.1
